# The Search of Association of HLA Class I and Class II Alleles with COVID-19 Mortality in the Russian Cohort

**DOI:** 10.3390/ijms24043068

**Published:** 2023-02-04

**Authors:** Valery Cheranev, Irina Bulusheva, Valery Vechorko, Dmitriy Korostin, Denis Rebrikov

**Affiliations:** Center for Precision Genome Editing and Genetic Technologies for Biomedicine, Pirogov Medical University, 1 Ostrovityanova Street, 117997 Moscow, Russia

**Keywords:** immunology, HLA-typing, COVID-19 mortality, diversity of HLA alleles, haplotype frequencies

## Abstract

HLA genes play a pivotal role in the immune response via presenting the pathogen peptides on the cell surface in a host organism. Here, we studied the association of HLA allele variants of class I (loci A, B, C) and class II (loci DRB1, DQB1, DPB1) genes with the outcome of COVID-19 infection. We performed high-resolution sequencing of class HLA I and class II genes based on the sample population of 157 patients who died from COVID-19 and 76 patients who survived despite severe symptoms. The results were further compared with HLA genotype frequencies in the control population represented by 475 people from the Russian population. Although the obtained data revealed no significant differences between the samples at a locus level, they allowed one to uncover a set of notable alleles potentially contributing to the COVID-19 outcome. Our results did not only confirm the previously discovered fatal role of age or association of DRB1*01:01:01G and DRB1*01:02:01G alleles with severe symptoms and survival, but also allowed us to single out the DQB1*05:03:01G allele and B*14:02:01G~C*08:02:01G haplotype, which were associated with survival. Our findings showed that not only separate allele, but also their haplotype, could serve as potential markers of COVID-19 outcome and be used during triage for hospital admission.

## 1. Introduction

In 2020, humankind was threatened by the epidemic that affected almost all countries to a certain degree. It was caused by a new virus from the Coronaviridae family—SARS-CoV-2 [1]. The first outbreak of the disease was registered in Wuhan (China), and then it rapidly spread all over the globe. By the moment this article has been prepared, the number of registered cases exceeded 650 million, over six million being lethal [2]. In January 2020, the World Health Organization (WHO) declared the outbreak to be a pandemic and termed the disease caused by SARS-CoV-2 virus as “COVID-19”.

One of the essential factors facilitating such a rapid spread of COVID-19 is a striking variability in the disease manifestations, as well as the mortality rate in individual patients [3,4,5]. The accumulated data on SARS-CoV-2 suggest correlation between the disease outcome and sex, age, and concurrent diseases [6]; the mortality rate can also be associated with individual genetic characteristics of a patient [7,8,9]. One of genetic predictors for the adverse disease outcome could be HLA class I and II genes encoding the proteins of the human leukocyte antigen (HLA).

The main function of these molecules is to present an antigen on the plasma membrane surface so that it would be recognized by immune cells. Therefore, the alleles encoding the amino acid sequences in HLA directly affect the susceptibility to certain diseases, e.g., H1N1 influenza (HLA-A*11, DRB1*10, HLA-B*35) [10], dengue infection (HLA-B*51, HLA-B*52, HLA-B*46) [11], AIDS (HLA-B*52) [12], etc. [13,14].

Based on the sequencing of the alleles of HLA class I and II genes and the protein composition of SARS-CoV-2, authors analyzed the affinity level of MHC binding to all possible viral epitopes [15,16]. The lowest predicted level of the interaction with viral antigens belonged to the protein encoded by B*46:01 allele, while the highest level belonged to the B*15:03 allele [15]. Another work, however, showed a negative correlation between the mortality rate and the frequency of HLA-DRB1*01:01 in the Mexican population (*n* = 26, R = −0.44, *p*-value = 0.02) [17]. The research on the influence of HLA genotype on COVID-19 severity revealed a significant difference in the allele frequency of HLA-DRB1*04:01 in severe patients as compared to the asymptomatic staff group in the European population (5.1% vs. 16.7%, *p*-value = 0.003 after adjustment for age and sex) [18]. In the research, which included the Russian population sample, mortality risk score was computed. As a result, the authors showed statistically significant correlations between the A*02:01 and A*03:01 alleles with a low mortality risk score and A*01:01 with a high mortality risk score [19].

Thus, HLA alleles may yield different results in terms of statistical significance depending on the studied population [7,8,17,18,20,21,22,23,24,25,26,27]. For instance, two large (n_1_ = 1980, n_2_ = 332) GWA studies produced quite opposite results: one of them failed to show any association between COVID-19 and HLA genes in European population [8], while the other one revealed three alleles (HLA-A*11:01, HLA-B*51:01, HLA-C*14:02) triggering the most severe disease outcomes in the Chinese population sample [7]. This variation in results may be related both to the specific frequencies of individual HLA alleles in populations and to the frequencies of the haplotypes they form. The loci A, B, C (class I) and loci DRB1, DQB1, and DPB1 (class II) of HLA genes are the most variable among all HLA genes, which determines their different affinity for the same antigen [15]. Furthermore, the linkage disequilibrium between alleles within a locus, as well as the haplotype frequencies within a population, may have a cumulative effect on antigen presentation [13,24].

In our work, we sequenced six HLA loci (class I (loci A, B, C) and class II (loci DRB1, DQB1, DPB1)) to compare the frequencies of HLA alleles and HLA haplotypes in three groups: (1) healthy donors from bone marrow registry, (2) patients who survived COVID-19 despite a severe course of a disease, and (3) patients who died from COVID-19 with an adjustment for age and comorbidities. This work aims to identify and validate the alleles significantly related to various COVID-19 outcomes. Therefore, we performed a retrospective analysis in order to reveal any possible associations between the identified alleles and the disease outcomes in the Russian population. First, differences in sex, age, and allele frequencies were assessed using Fisher’s exact test and Pearson’s chi-squared test. Then, Hardy-Weinberg equilibrium and nonequilibrium linkage were evaluated for each group and each locus. After that, haplotype frequencies were assessed using the maximum likelihood method, and differences in frequencies were evaluated using Fisher’s exact test and the t-test. In the final step, logistic regression models, including sex, age, and HLA locus or allele, were constructed.

## 2. Results

### 2.1. The Age and Sex Distribution in the Studied Samples

**Figure 1:**
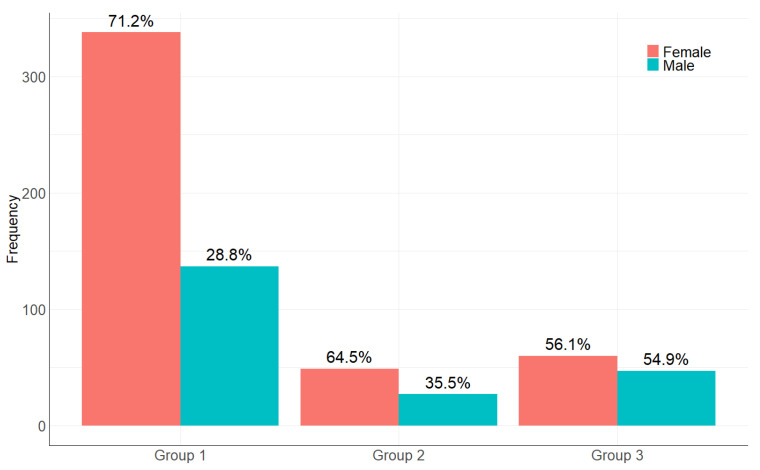
Sex distribution in the studied samples.

Females predominated in all studied groups (Figure 1).

Using the Pearson’s chi-squared test, we detected a statistically significant deviation between the groups (Table 1).

Pairwise comparisons revealed statistically significant differences between groups 1 and 3 (Table 2). In group 2B, the number of males (16.67%) was statistically significantly lower compared to the other selected age groups (from 39.19% to 54.5%). We did not detect any significant differences between the other groups.

**Figure 2:**
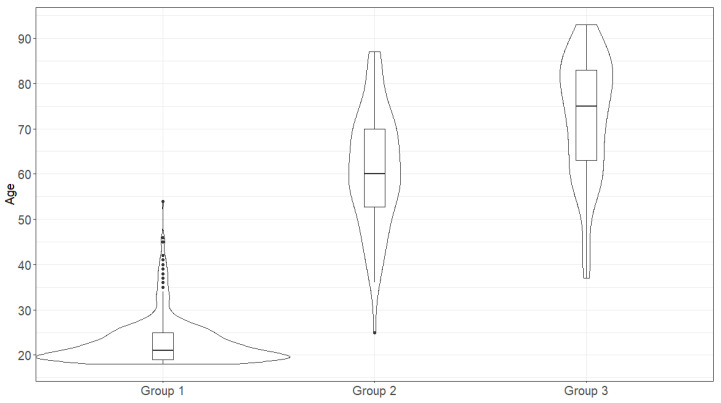
Age distribution in the studied samples.

The age distribution in groups is shown in Figure 2. Only in the second group did we observe a distribution that does not differ from normal (Shapiro-Wilk test, *p* = 0.72). In the other groups, there was a marked shift toward older age (group 3, Shapiro-Wilk test, *p* < 0.05) or younger age (group 1, Shapiro-Wilk test, *p* < 0.05). For this reason, the non-parametric Mann-Whitney test was chosen as the statistical test.

The statistically significant differences in the age were observed in all compared groups (Table 3).

The average age of deceased patients homozygous for at least one allele of class I loci (A, B, C) was lower as compared to the patients without homozygous loci (the Mann-Whitney U test; *p*-value < 0.05). At the same time, we did not detect any statistically significant differences in the age of patients homozygous for class I or class II loci, class II loci (in combinations), or homozygous for at least one locus.

### 2.2. The Distribution of Allele Frequencies, the Hardy-Weinberg Equilibrium and the Influence of the Gene Linkage Disequilibrium in the Studied Samples

The data obtained from high-resolution HLA typing for each patient included the information on both alleles of A*, B*, C*, DRB1*, DQB1*, and DPB1* loci of genes in the HLA histocompatibility complex (see HLA genotypes of all groups in Supplementary Table S1). Each allele of every gene that exhibited significant differences in the allele frequencies on the locus and allele level was analyzed in detail. The distribution of allele frequencies over six loci from three groups is shown in Supplementary Figure S1a–f.

The Hardy-Weinberg equilibrium is disrupted in the group 1 for the HLA-A locus and in the group 3 for the HLA-B and HLA-C loci (Table 4).

The linkage disequilibrium was more prominent in group 1. All analyzed loci in this group were related statistically significant to each other. In group 2, the linkage between the HLA-A and HLA-DPB1 (*p*-value = 0.12), as well as HLA-C and HLA-DPB1 (*p*-value = 0.51), was statistically insignificant. In group 3, statistically insignificant linkage was observed only between the following pairs: HLA-A and HLA-DQB1 (*p*-value = 0.25), HLA-A and HLA-DPB1 (*p*-value = 0.88), and HLA-C and HLA-DPB1 (*p*-value = 0.08). Based on the obtained results, we further performed a haplotype analysis for all six loci (A, B, C, DRB1, DQB1, DPB1), as well as for five loci (A, B, C, DRB1, DQB1), for the class I and II loci, and the pairs of the HLA-B and HLA-C demonstrating statistically significant deviations from the Hardy-Weinberg equilibrium.

### 2.3. The Distances upon Pairwise Comparisons

The data from three groups were analyzed using the method of distances upon pairwise comparisons [28] by the fixation index F_st_ [29] (Table 5). Zero and negative values of F_ST_ usually indicate the absence of genetic stratification between the populations, while positive values show the presence of differences.

Results obtained from the comparison of genotype probability graphs of each sample population were consistent with the conclusions from the pairwise comparison, all group pairs exhibiting similar or at least slightly different behavior. We observed no statistically significant differences between the groups.

### 2.4. Estimation of the Allele Distribution at Locus and Allele Levels

We applied the Pearson’s goodness-of-fit test to each HLA locus to estimate the significance of allele distribution. A separate analysis of the three groups showed significant effects at a locus level (Table 6), except for the DQB1 locus analyzed by the V1 method. The analysis of the combined groups (1 + 2 vs. 3: healthy donors or recovered patients vs. patients died from COVID-19) by both methods did not reveal any notable difference at the locus level.

At the next stage, we applied the Pearson’s goodness-of-fit test to each allele (V2) or allele combination (V1) from three groups.

To identify significant alleles, we studied the combinations of other groups using the V1 and V2 methods. We analyzed different group combinations to determine significant individual alleles or their pairs associated with either a good or bad outcome (Table 7 and Table S1). The direct comparative analysis of group 2 and 3 revealed no significant difference in allele frequencies, and neither did the comparison of different ages across the groups.

Finally, various combinations of groups were studied to determine significant alleles (the list of combinations in the Supplementary Table S7). Of note, the most interesting findings were represented by the difference of the DQB1*05:03 allele frequencies between groups 1 and 3.

The Fisher’s exact test allowed for identifying 14 statistically significant alleles (Supplementary Table S8). However, only the HLA-DQB1*05:03:01G allele showed statistical significance after the multiple comparison correction (Holm-Bonferroni).

### 2.5. Comparison of Haplotype Frequencies

The most frequent haplotypes estimated for six loci showed significant differences between the samples (Supplementary Table S2). For instance, the haplotype including the A*01:01:01G~B*08:01:01G~C*07:01:01G~DRB1*03:01:01G~DQB1*02:01:01G alleles exhibited similar frequencies across the groups (Group 1–2.5% ± 0.6, Group 2–3.3% ± 1.5, Group 3–2.8% ± 1.0). However, these haplotypes contained different alleles in DPB1 locus (Group 1 and Group 3 contained DPB1*04:01:01G, Group 2 included DPB1*01:01:01G). Another frequent haplotype was A*02:01:01G~B*13:02:01G~C*06:02:01G~DRB1*07:01:01G~DQB1*02:01:01G, which also had similar frequencies across different groups (Group 1–2.2% ± 0.5, Group 2–1.97% ± 1.3, Group 3–3.2% ± 1.1), but contained different alleles in the DPB1 locus (Group 1 and Group 3-DPB1*04:01:01G, Group 2–DPB1*17:01:01G). Group 2 had the A*03:01:01G~B*13:02:01G~C*06:02:01G~DRB1*07:01:01G~DQB1*02:01:01G~DPB1*04:01:01G haplotype with a distinct HLA-A locus (A*03:01:01G instead of A*02:01:01G). Furthermore, the differences in the DPB1*17:01:01G allele between groups 1 and 2 were statistically significant as determined by the Fischer’s exact test without multiple comparison correction. Since the significant linkage disequilibrium between the HLA-A and HLA-DPB1 loci in groups 2 and 3 was absent, haplotype analysis based on six loci by maximal likelihood was more likely to provide the error-prone results, especially in case of small sample sizes. Therefore, we repeated the haplotype analysis using five loci (A, B, C, DRB1, DQB1), those being only the class I loci and class II loci. We also analyzed the HLA-B and HLA-C pairs separately, as they exhibited disrupted Hardy-Weinberg equilibrium in group 3.

The most frequent haplotypes estimated for five loci showed a higher convergence as compared to the analysis of six loci (Supplementary Table S3). The most frequent haplotype, A*01:01:01G~B*08:01:01G~C*07:01:01G~DRB1*03:01:01G~DQB1*02:01:01G, was identical in all studied groups (Group 1–4.7% ± 0.7, Group 2–3.95% ± 1.5, Group 3–5.1% ± 1.2), the differences in frequencies being statistically insignificant. Comparison of the haplotype frequencies between groups 2 and 3 showed statistically significant differences only for one haplotype (Table 8, Haplotype 2). This haplotype was frequently detected in group 1 (~0.95%, sd ± 0.34) and group 2 (~3.29%, sd ± 1.5). However, in group 3, it was registered only once. Statistical analysis of the allele frequencies by the exact Fischer’s test showed that lower frequency of alleles from this haplotype in group 3 was statistically significant. However, the significance was not detected after the multiple comparison corrections (Table S4). Comparing the frequencies of the haplotypes between group 1 and 2 did not reveal any statistically significant differences. Comparing group 1 and 3 resulted in identifying the (A*02:01:01G~B*27:02:01G~C*02:02:02G~DRB1*16:01:01G~DQB1*05:02:01G) haplotype, which had a statistically significantly higher occurrence in the group of deceased patients (exact Fisher’s test *p* < 0.05, *t*-test adjusted *p* < 0.05).

Analyzing class I haplotypes (Supplementary Table S4) revealed only one haplotype (A*02:01:01G~B*27:02:01G~C*02:02:02G. The differences in frequencies between the groups (Group 3–2.2% ± 0.87; Group 1–0.3% ± 0.19) were shown to be statistically significant by two tests (exact Fisher’s test *p*-value = 0.003, *t*-test adjusted *p*-value = 0.028). 

Comparing group 2 and 3 (Supplementary Table S5) enabled identifying two haplotypes (DRB1*01:01:01G~DQB1*05:01:01G~DPB1*04:02:01G (Group 2–4.8% ± 1.8; Group 3–1.1% ± 0.8); DRB1*07:01:01G~DQB1*02:01:01G~DPB1*17:01:01G (Group 2–5.9% ± 1.8; Group 3–1.5% ± 0.7)), the differences in their frequencies between the groups being statistically significant (exact Fisher’s test *p* < 0.05, *t*-test adjusted *p* < 0.05; exact Fisher’s test *p* < 0.05, *t*-test adjusted *p* < 0.05). Comparing group 1 and 2 allowed us to single out the haplotype (DRB1*07:01:01G~DQB1*02:01:01G~DPB1*17:01:01G) with a frequency (Group 1–1.5% ± 0.4; Group 2–5.9% ± 2.0) exhibiting statistically significant differences between the groups (exact Fisher’s test *p* < 0.05, *t*-test adjusted *p* < 0.05). Comparison of the frequencies of the class II loci between groups 1 and 3 revealed the haplotype (DRB1*11:01:01G~DQB1*03:01:01G~DPB1*04:02:01G). Its frequency was statistically significant between the groups (Group 1–0.6% ± 0.4; Group 3–2.9% ± 1.0), according to the results of two tests (exact Fisher’s test *p* < 0.05, *t*-test adjusted *p* < 0.05).

Comparing the frequencies of the HLA-B and HLA-C loci (Supplementary Table S6) between groups 2 and 3 allowed identifying two haplotypes (B*14:02:01G~C*08:02:01G (Table 8, Haplotype 4) (Group 2–4.6% ± 1.7; Group 3–1.2% ± 0.6); B*57:01:01G~C*06:02:01G (Group 2–5.9% ± 1.8; Group 3–1.9% ± 0.8)) with statistically significantly different frequencies (exact Fisher’s test *p* < 0.05, *t*-test adjusted *p* < 0.05; exact Fisher’s test *p* < 0.05, *t*-test adjusted *p* < 0.05). Analyzing group 1 and 2 revealed the haplotype (B*57:01:01G~C*06:02:01G (Group 1–2.3% ± 0.5; Group 2–5.9% ± 1.89)) with a frequency being statistically significantly different between groups (exact Fisher’s test *p*-value = 0.029, *t*-test adjusted *p*-value = 0.0078). Comparing group 1 and 3 revealed the haplotype (B*27:02:01G~C*02:02:02G (Group 1–0.8% ± 0.29; Group 3–2.5% ± 0.89)) with statistically different frequencies between groups (Exact Fisher’s test *p* < 0.05, *t*-test adjusted *p* < 0.05).

### 2.6. Logistic Regression

First, for logistic regression, we excluded the HLA loci and used only patients’ sex, age, and their interaction. Statistical significance was observed for the intercept (*p* < 0.05) and age (*p* < 0.05) (Table 9).

In order to avoid the effect of linkage disequilibrium and, as a consequence, the presence of strongly correlated independent variables, each locus was analyzed independently. 

In our logistic model, including only the locus HLA-A alleles, revealed statistical significance of the A*33:01:01G allele (*p* < 0.05), intercept (*p* < 0.05), and age (*p* < 0.05). We selected the best model by gradually excluding the predictors based on the AIC parameter and observed a loss of statistical significance by the allele.

Using the logistic model based only on the HLA-B locus alleles, observed, statistical significant predictors included HLA-B*14:02:01G (*p* < 0.05), HLA-B*35:03:01G (*p* < 0.05), HLA-B*38:01:01G (*p* < 0.05), HLA-B*40:01:01G (*p* < 0.05), HLA-B*57:01:01G (*p* < 0.05) alleles, and age (*p* < 0.05). After selecting the best model by gradually excluding the predictors based on the AIC parameter, along with the predictors mentioned above, we identified significant differences in the frequencies of HLA-B*07:02:01G (*p* < 0.05), HLA-B*13:02:01G (*p* < 0.05), HLA-B*18:01:01G (*p* < 0.05), HLA-B*41:01:01G (*p* < 0.05), HLA-B*44:03:01G (*p* < 0.05), and HLA-B*56:01:01G (*p* < 0.05). A separate model, including a single allele, sex, and age, was statistically significant only in case of the HLA-B*14:02:01G allele (*p* < 0.05).

The logistic model including only the HLA-C locus alleles demonstrated statistical significance only for the intercept (*p* < 0.05), age (*p* < 0.05), and the HLA-C*08:02:01G allele (*p* < 0.05). After selecting the model by gradually excluding the predictors based on the AIC parameter, we identified additional significant predictors: HLA-C*12:02:01G, HLA-C*15:02:01G, and the interaction of sex and age (Table 10). The model, including sex, age, and the HLA-C*08:02:01G allele, also displayed statistical significance (Table 11). The model including only the HLA-C*12:02:01G or C*15:02:01G allele did not show any statistical significance except age or the intercept.

The logistic model based only on the HLA-DRB1 locus alleles did not reveal any statistically significant determinants apart from the age. After selecting models by gradually excluding the predictors based on the AIC parameter, the statistical significance was shown for the HLA-DRB1*01:01:01G, HLA-DRB1*01:02:01G alleles (Table 12). The model including age, sex, and the HLA-DRB1*01:01:01G allele also demonstrated statistically significant results (*p* < 0.05) (Table 13). The model based on age, sex, and the DRB1*01:02:01G allele did not confirm its statistical significance.

The logistic model including only the HLA-DQB1 locus alleles did not reveal statistically significant determinants apart from the age. After selecting models by gradual exclusion of predictors based on the AIC parameter, statistical significance was detected in case of the HLA-DQB1*05:01:01G allele (Table 14). The model based on sex, age, and the HLA-DQB1*05:01:01G allele was also found to be statistically significant (Table 15). 

The logistic model including the DPB1 locus alleles did not reveal any statistically significant differences, except the age, for all alleles in this locus as well as for individual alleles.

We also designed the model based on all identified statistically significant alleles HLA-C*08:02:01G (HLA-B*14:02:01G was removed due to 100% correlation with HLA-C*08:02:01G), HLA-DRB1*01:01:01G, and HLA-DQB1*05:01:01G, as well as sex, age, and their interaction. After selecting the models by gradually excluding the predictors based on the AIC parameter, statistically significant predictors included only DRB1*01:01:01G and HLA-C*08:02:01G (Table 16).

## 3. Materials and Methods

### 3.1. Subjects

The group of recovered patients (group 2) with severe symptoms, and the patients who died from COVID-19 (group 3) were divided into subgroups (2A, 2B, 3A, 3B) according to the age (age < 65 or ≥ 65 at the moment of death/illness). Clinical features of the groups are presented in Table 17, Table 18, Table 19 and Table 20, except 50 members from group 3 with no data other than HLA genotypes and the outcome. As a control sample population, we used 475 venous blood samples collected from the members of the National Registry of Bone Marrow Donors at the Pirogov Medical University in the beginning of 2020.

### 3.2. Biomaterial Collection

The exploited biomaterial consisted of venous whole blood collected into EDTA-coated tubes. Diagnostic criteria for inclusion to the study were fever and/or respiratory symptoms and the positive test for COVID-19 was confirmed by RT-qPCR test (to estimate viral RNA content)–named «SARS-CoV-2/SARS-CoV» (DNA Technology, Russia)—from nasopharyngeal swabs in Moscow clinical diagnostic laboratories that collected the biomaterial. Patients with pathologies that led to greater morbidity or who had additional immunosuppression (patients with HIV, active cancer in treatment with chemotherapy, immunodeficiency, autoimmune diseases with immunosuppressants, and transplants) were not included in the study.

### 3.3. gDNA Isolation, Library Preparation and Sequencing

gDNA was isolated from 100 uL of venous whole blood with the Proba-McheMaks (DNA Technology LLC, Moscow, Russia) reagent kit using the automated dosing station DTstream (DNA Technology LLC, Moscow, Russia). This method involved a routine step including lysis, DNA precipitation on magnet beads, three washing steps, and an elution step. Quality control of the isolated DNA was performed by agarose gel electrophoresis; the concentration was measured using Qubit 3 fluorometer with Qubit dsDNA BR Assay kit (ThermoFisher Scientific, Grand Island, NY, USA) (mean concentration—31.03 ng/uL, standard deviation—50.28 ng/uL, median–15.2 ng/uL, range–1.01–200 ng/uL).

The preparation of amplicon libraries for HLA high-resolution genotyping was performed using HLA Expert kit (DNA Technology LLC, Moscow, Russia) following the manufacturer’s protocol (Kit was certified by Russian Federal Service for Surveillance in Healthcare (Roszdravnadzor)). It included several steps. The first stage involved a qPCR for human gene that does not have pseudogenes and is presented in a single copy. This was required for the estimation of a concentration and the presence of inhibitors in a genomic DNA sample. The results were used for normalization of DNA amount during the following step. The second stage involved a multiplex PCR for most variable exons (2, 3, 4 for the HLA class I and 2, 3 for the HLA class II). Primers were designed using conservative regions of gene introns flanking the exons. Several primers with one nucleotide shift were used to prevent an imbalance in nucleotide content during sequencing. The third stage involved ligation of the adapters containing Illumina i5 and i7 indexes. The fourth stage was an additional routine PCR (6 cycles) with the p5 and p7 primers. The purification with magnetic beads (SPRI type) was performed after each stage. Quality control of the libraries was performed using agarose gel electrophoresis; the concentration was measured using the Qubit 3 fluorometer with the Qubit dsDNA HS Assay kit (ThermoFisher Scientific, USA).

Sequencing was performed using the Illumina MiSeq platform (Illumina, San Diego, CA, USA) with the MiSeq Reagent Kit v3 (600-cycle), according to the manufacturer’s protocol.

Fastq files were analyzed with HLA-Expert software (DNA Technology LLC, Moscow, Russia) following the manufacturer’s instructions. Obtained exon sequences were aligned to the human major histocompatibility complex (MHC) sequences IMGT/HLA v3.41.0 [30]. 

Basic quality control metrics for QC included:

- Quality threshold for reads (low quality reads were trimmed or discarded);
- Lowest absolute and relative coverage for each position;
- The highest number of differences (insertions, substitutions, deletions) from the group average for each read;
- Maximum relative position error—the number of differences (insertions, substitutions, deletions) from the consensus sequence in each position should not exceed the specified threshold;
- The highest average error per read for a group;
- The lowest number of reads in groups for each exon (I-class 2,3,4 exons, II-class-2,3 exons);
- The allelic imbalance should not exceed a given threshold; the ratio of the read number for the exons from each allele and the sum of these ratios;
- The presence of phantom (cross-mapping) and chimeric sequences;
- The percentage of combined, clustered, and used for typing reads computed for each sample.

### 3.4. Statistical Analysis

Allele frequencies in the analyzed cohorts were estimated by dividing the number of occurrences of a given allele in an individual by the doubled total number of individuals (alleles of homozygous individuals were counted as two occurrences). Statistical analysis included the Pearson’s goodness-of-fit test (for the distribution of alleles in each HLA-locus, allele and allele combination, sex ratios in groups), the Fisher`s exact test for determining the significances in differences between allele frequencies, the Wilcoxon rank sum test with continuity correction for estimating the differences in age between all groups. Arlequin (version 3.5.2.2) was used to conduct population assignment test, estimate the Hardy-Weinberg equilibrium, pairwise linkage disequilibrium, and measure the distances upon pairwise comparisons between all three groups [31]. We created several scripts in order to estimate the diversity of each gene and differences in the frequencies of individual alleles. Another script we had designed was aimed at correcting an input table with patients’ data and transforming the names of HLA alleles following a unified syntax (https://github.com/genomecenter/HLA_article; accessed on 22 June 2021). We created a script that generated an input file containing patients’ data for Arlequin.

In order to determine the significant alleles for each gene by compiling a contingency matrix, we used the Holm-Bonferroni method [32] with the significance level of 0.05 for multiple comparison correction. For that purpose, we designed a special script. Haplotype frequencies were estimated by Arlequin (version 3.5.2.2) using the expectation-maximum algorithm. Haplotype frequencies were determined for the class I loci, class II loci, 5 loci (A, B, C, DRB1, DQB1), and 6 loci (A, B, C, DRB1, DQB1, DPB1). The standard deviation was assessed by bootstrapping (*n* = 1000). The differences of mean frequencies of haplotypes between samples were compared with the t-test with mean haplotype frequencies and standard deviation (the number of identified haplotypes in samples were used as freedom degrees). After that, we checked the results by the Fisher’s exact test.

We employed the Pearson’s goodness-of-fit test to study each gene separately and analyzed the 2-field level of alleles for each gene. The null hypothesis stated that HLA did not affect the divergence of allele distribution and allele frequencies in the groups. For evaluating the role of single alleles and allele combinations, they were selected from the groups by two methods. The contingency matrix for each gene was compiled using one of two methods (Table 21). The first method employed allele combinations (both alleles in a pair) to produce a contingency table, which allows for estimating the significance of the impact of an allele combination present in each locus on the disease outcome. The second method–approximating to biological processes in organisms–enabled computing the allele carrier in each group.

We used logistic regression to discern the impacts of age and sex from the influence of alleles. We employed two models. The first model included only sex and age as independent variable and an outcome as a dependent variable. The second one used sex, age, and the presence of a certain allele as an independent variable and an outcome as a dependent variable. Analysis was conducted with glm and step from the R stats package (ver 4.2.0).

### 3.5. Used Formulas

Fisher’s exact test (R language):

$$p=\frac{\left(a+b\right)!\left(c+d\right)!\left(a+c\right)!\left(b+d\right)!}{a!\;b!\;c!\;d!\;n!}$$

Pearson’s chi-square test (Python 3.9 language):

$$\lambda^{2}=\sum \frac{{\left(\left|O-E\right|-0.5\right)}^{2}}{E}$$

Unequal variances t-test (R language):

$$t=\frac{{\bar{X}}_{1}-{\bar{X}}_{2}}{s_{\bar{\Delta }}}$$

Haplotype frequency estimation (*D*—probability function, *p*—given haplotype frequencies) (Arlequin):

$$L\left(D|p\right)=\overset{n}{\underset{i=1}{\sum }}\overset{g_{i}}{\underset{j=1}{\prod }}G_{ij}$$

Logistic regression formula (R language):

$$\mathrm{Outcome}\;\sim \;\mathrm{Age}*\mathrm{Sex}+{\mathrm{HLA}}_{\mathrm{loci}}/\mathrm{HLA}\_\mathrm{allele}$$

## 4. Discussion

Since the HLA genotype determines an individual repertoire of immune response to foreign pathogens, it could contribute to COVID 19 susceptibility and severity. Particularly, it is of importance to analyze certain alleles and haplotype frequencies in detail across different populations.

Several studies (see Table 5) suggested a number of alleles that might be statistically significant to predict a possible COVID-19 outcome. Different methods of computing significance showed specific for population results. Analysis of HLA protein affinity [15,16] showed that B*46:01 and C*12:03 or C*14:02 and A*02:01 alleles have a statistically significant association with COVID-19. At the same time, comparing the mortality level and the diversity of HLA alleles [17,20,27] allowed us to suggest the existence of other associated alleles (DRB1*01:01, C*05, A*02:01).

In our work, we scrutinized three groups comprising the representatives of the Russian population. We used various approaches for estimating the link between individual HLA alleles and generated haplotypes with the COVID-19 outcome. We found a significant bias in the sex and age composition of the population. Group 1 mostly consisted of females (71.2%), whereas group 3 included mostly males (56.1%). This may be explained by the statistically significantly association of the sex with the survival rate after severe disease as it was shown previously [1]. To the contrast, group 2B included fewer males (16.67%). Age is the significant factor, which was confirmed in our work, as well as in earlier works [1]. Estimating the Hardy-Weinberg equilibrium showed the disruption of an equilibrium in the HLA-B and HLA-C loci, which, together with the linkage disequilibrium, drew our attention to these loci. The list of all alleles that were statistically significantly associated with the disease outcomes is shown in the Table. Employing the first method of creating contingency tables (V1), we identified the statistically significant differences in the numbers of patients with the HLA-A*01:01:01G allele homozygosity between groups 3A and 3B (*p* < 0.05), group 1 and group 3A (*p* < 0.05), which is in line with the previous results [19]. However, after the multiple comparison corrections, the differences became statistically insignificant and were not detected upon comparison of groups 2 and 3. Estimating the mean age in group 3 showed that, on average, patients with class I allele homozygosity died more frequently, which also keeps up with the previous data [19]. Meanwhile, in group 2 or loci belonging to the other class, these did not display a similar association, implying a greater contribution of the class I loci to the disease severity.

Our findings reveal that due to linkage disequilibrium, the statistically significant alleles were combined into individual haplotypes that could predominate in the populations of deceased patients (B*27:02:01G~C*02:02:02G) or survivors (B*14:02:01G~C*08:02:01G). The obtained results are only partially consistent with the previous findings (DRB1*01:01, DRB1*01:02), which can be related to the population characteristics, as well as with the insufficient sample size that greatly decreased the statistical power of the analysis. Another decline in the analysis efficiency might arise from the possibility that, in the population, the alleles with high affinity toward viral peptide (C*08:02:01G, A*02:01:01G, C*02:02:02G) can be co-inherited with the alleles showing the lower affinity (B*14:02:01G, B*27:02:01G) (Ref. [15]). Except for DRB1*01:01:01G and DRB1*01:02:01G, none of the alleles or haplotypes that were statistically significant in our study were identified in other works (Table 22). This may be due to the specificity of the population frequencies of the alleles and haplotypes, which directly affects the power and possibility of applying statistical methods.

The main limitation of this work is the lack of detailed clinical data for patients, which does not allow for a study of the relationship between the analysis of HLA genotype or haplotypes and additional factors affecting survival, such as comorbidities, BMI, etc. Another important limitation is that the relatively small sample size, which could significantly reduce the power of the assessment methods used, especially for rare alleles and haplotypes.

## 5. Conclusions

In the present study, we analyzed HLA genotypes of three Russian population samples: healthy individuals, patients who survived severe COVID-19, and patients who died from it. Using the Fisher’s exact test and Pearson’s goodness-of-fit test, we performed haplotype frequencies analysis an logistic regression to show that the alleles of loci A, B, C, DRB1, DQB1 and DPB1 influenced the COVID-19 outcome. The immediate results of the research showed the absence of any significant difference between the groups at the locus level, however, several alleles proved to be perspective. These embrace the already known DRB1*01:01 and DQB1*05:03, detected in the current research, since they presumably influence the outcome of COVID-19. We also found a decrease in the frequency of one of the common haplotypes (B*14:02:01G~HLA-C*08:02:01G) in the group of deceased patients. On the contrary, the frequency of this haplotype in the group of survivors three times exceeded its occurrence in the control group. Still, the results allow us to conclude that the associations of HLA alleles with COVID-19 progression and outcome depend largely on individual characteristics of the population under investigation. In further work, we plan to collect samples of larger size and more detailed information on comorbidities, which will allow to obtain higher power for statistical criteria, as well as to make a more accurate assessment of the role of HLA genes and their haplotypes in the course of the disease.

## Figures and Tables

**Table 1:** Sex distribution in the studied samples.

Sex	Group 1	Group 2	Group 3	
Female	338 (71.2%)	49 (64.5%)	60 (56.1%)	χ^2^ = 9.6, df = 2, *p*-value < 0.05
Male	137 (28.8%)	27 (35.5%)	47 (43.9%)	

**Table 2:** Sex distribution in the studied groups.

Group Combination	Pearson’s Goodness-of-Fit Test Result
Group 1 + Group 2	χ^2^ = 1.1, df = 1, *p*-value = 0.3
Group 1 + Group 3	χ^2^ = 8.51, df = 1, *p* < 0.05
Group 2 + Group 3	χ^2^ = 0.98, df = 1, *p*-value = 0.3
Group 2A + Group 2B	χ^2^ = 6.4, df = 1, *p* < 0.05
Group 2A + Group 3A	χ^2^ = 0.13, df = 1, *p*-value = 0.7
Group 2A + Group 3B	χ^2^ = 0.55, df = 1, *p*-value = 0.46
Group 2B + Group 3A	χ^2^ = 8.17, df = 1, *p* < 0.05
Group 2B + Group 3B	χ^2^ = 3.95, df = 1, *p* < 0.05
Group 3A + Group 3B	χ^2^ = 1.67, df = 1, *p*-value = 0.2

**Table 3:** Difference in age distribution in the studied samples.

Group Combination	Median Values in Compared Groups		The Wilcoxon Rank Sum Test with Continuity Correction
	First Group	Second Group	
Group 1 + Group 2	21	60	W = 193, *p* < 0.05
Group 1 + Group 3	21	75	W = 50,775, *p* < 0.05
Group 2 + Group 3	60	75	W = 2122.5, *p* < 0.05
Group 2A + Group 3A	71.5	80.5	W = 573, *p* < 0.05
Group 2B + Group 3B	56	58	W = 559.5, *p* < 0.05

**Table 4:** Hardy-Weinberg equilibrium in studied samples.

Locus	Group 1			
	The Number of Genotypes	Observed Heterozygosity	Expected Heterozygosity	*p*-Value
HLA-A	475	0.897	0.88	*p* < 0.05
HLA-B	475	0.96	0.95	0.09
HLA-C	475	0.91	0.91	0.42
HLA-DRB1	475	0.94	0.93	0.67
HLA-DQB1	475	0.88	0.86	0.5
HLA-DPB1	475	0.75	0.75	0.9
	Group 2			
HLA-A	76	0.91	0.87	0.63
HLA-B	76	0.96	0.95	0.66
HLA-C	76	0.89	0.91	0.39
HLA-DRB1	76	0.93	0.91	0.94
HLA-DQB1	76	0.87	0.87	0.41
HLA-DPB1	76	0.789	0.798	0.94
	Group 3			
HLA-A	157	0.87	0.86	0.3
HLA-B	157	0.96	0.95	*p* < 0.05
HLA-C	157	0.92	0.90	*p* < 0.05
HLA-DRB1	157	0.94	0.92	0.06
HLA-DQB1	157	0.86	0.87	0.34
HLA-DPB1	157	0.83	0.77	0.78

**Table 5:** F_st_ distance between groups.

	Group 1	Group 2	Group 3
Group 1	-		
Group 2	0.00086 (*p*-value = 0.14)	-	
Group 3	−0.00003 (*p*-value = 0.44)	0.00075 (*p*-value = 0.2)	-

**Table 6:** Comparison of all three groups using the Pearson’s goodness-of-fit test.

Locus	Selection Principle	Chi-Square	Freedom Degrees	*p*-Value
HLA-A	V1	254.4	272	0.77
	V2	48.2	64	0.93
HLA-B	V1	644.5	644	0.49
	V2	124.7	106	0.10
HLA-C	V1	286.1	290	0.55
	V2	37.7	46	0.91
HLA-DRB1	V1	417.4	430	0.66
	V2	73.0	72	0.44
HLA-DQB1	V1	214.9	168	*p* < 0.05
	V2	37.3	32	0.24
HLA-DPB1	V1	164.8	158	0.34
	V2	61.1	48	0.1

**Table 7:** Comparison of group combinations analyzed by V4 method.

Group Combination	Method	HLA Locus	Significant Allele (Number, Group)	Chi-Square, df, *p*-Value	Adj *p*-Value (Holm-Bonf)
**1 + 2 + 3**	V1	A*	23:01_24:02 (*n* = 2, group 2; *n* = 0, group 1, group 3)	16.7, 2, *p* < 0.05	*p* < 0.05
	V2	DQB*	05:03:01G (*n* = 8, group 1; *n* = 8, group 2; *n* = 7, group 3)	13.9, 2, *p* < 0.05	*p* < 0.05
**1 + (2 + 3)**	V2	DQB*	05:03:01G (*n* = 8, group 1; *n* = 15, group 2 + 3)	9.8, 1, *p* < 0.05	*p* < 0.05
**1 + 2**	V2	DQB*	05:03:01G (*n* = 8, group 1; *n* = 8, group 2)	11.2, 1, *p* < 0.05	*p* < 0.05
**1 + 3A**	V1	B*	07:02_13:02 (*n* = 7, group 3A; *n* = 3, group 1)	23.2, 1, *p* < 0.05	*p* < 0.05
	V2	B*	27:02 (*n* = 8, group 3A; *n* = 24, group 1)	11.6, 1, *p* < 0.05	*p* < 0.05
**1 + 3B**	V1	C* DQB1*	12:03_15:02 (*n* = 3, group 3B; *n* = 3, group 1) 03:03_06:03 (*n* = 2, group 3B; *n* = 0, group 1)	12.8, 1, *p* < 0.05 16.0, 1, *p* < 0.05	*p* < 0.05 *p* < 0.05

**“+”** separates the compared groups; parentheses indicate groups merged for comparison.

**Table 8:** The number of haplotypes per group.

№	Haplotype	Group Number							Fisher’s Exact Test	
		1	2	2A	2B	3	3A	3B	Group Combination	*p*-Value
1	A*33:01:01G~ B*14:02:01G~ C*08:02:01G~ DRB1*01:02:01G~ DQB1*05:01:01G~ DPB1*04:01:01G	9	5	3	2	1	1	0	Group 1 + Group 2 Group 1 + Group 3 Group 2 + Group 3	*p* < 0.05 0.47 *p* < 0.05
2	A*33:01:01G~ B*14:02:01G~ C*08:02:01G~ DRB1*01:02:01G~ DQB1*05:01:01G	11	5	3	2	1	1	0	Group 1 + Group 2 Group 1 + Group 3 Group 2 + Group 3	0.06 0.31 *p* < 0.05
3	A*33:01:01G~ B*14:02:01G~ C*08:02:01G	14	5	3	2	2	2	0	Group 1 + Group 2 Group 1 + Group 3 Group 2 + Group 3	0.17 0.14 *p* < 0.05
4	B*14:02:01G~ C*08:02:01G	24	7	4	3	3	3	0	Group 1 + Group 2 Group 1 + Group 3 Group 2 + Group 3 Group 2A + Group 3A	0.18 0.17 *p* < 0.05 0.2

**Table 9:** Coefficients of model without HLA loci.

	Dependent Variable: Outcome
Age	−0.06 *** (0.02)
SexMale	1.87 (2.01)
Age:SexMale	−0.04 (0.03)
Constant	3.9 *** (1.14)
Observation	183
Log Likelihood	−97.19
Akaike Inf. Crit	208.38

Note: *** *p* < 0.01.

**Table 10:** Coefficients for “Best” model by AIC for locus C.

	Dependent Variable: Outcome
Age	−0.08 *** (0.06)
SexMale	−0.97 *** (0.38)
HLA-C*08:02:01G-carriage	1.64 ** (0.78)
HLA-C*12:02:01G-carriage	−2.47 ** (1.25)
HLA-C*15:02:01G-carriage	−1.8 * (0.99)
HLA-C*16:02:01G-carriage	15.1 (996.86)
Constant	5.51 *** (1.07)
Observation	183
Log Likelihood	−97.19
Akaike Inf. Crit	208.38

Note: * *p* < 0.1; ** *p* < 0.05; *** *p* < 0.01.

**Table 11:** Coefficients for one-allele (HLA-C*08:02:01G) model.

	Dependent Variable: Outcome
Age	−0.06 *** (0.02)
SexMale	2.26 (2.09)
HLA-C*08:02:01G-carriage	1.87 ** (0.81)
Age:SexMale	−0.05 (0.03)
Constant	3.94 *** (1.15)
Observation	183
Log Likelihood	−101.58
Akaike Inf. Crit	213.16

Note: ** *p* < 0.05; *** *p* < 0.01.

**Table 12:** Coefficients for “Best” model for locus DRB1.

	Dependent Variable: Outcome
Age	−0.09 *** (0.02)
SexMale	−1.27 *** (0.42)
HLA-DRB1*01:01:01G-carriage	1.05 ** (0.48)
HLA-DRB1*01:02:01G-carriage	2.52 ** (1.24)
HLA-DRB1*04:05:01G-carriage	−20.16 (3956.18)
HLA-DRB1*08:04:01G-carriage	−18.68 (3956.18)
HLA-DRB1*09:01:02G-carriage	−17.64 (1574.57)
HLA-DRB1*12:01:01G-carriage	1.31 (0.84)
HLA-DRB1*14:03:01G-carriage	−18.64 (3956.18)
HLA-DRB1*14:05:01G-carriage	19.08 (3956.18)
Constant	6.11 *** (1.2)
Observation	183
Log Likelihood	−90.14
Akaike Inf. Crit	202.28

Note: ** *p* < 0.05; *** *p* < 0.01.

**Table 13:** Coefficients for one-allele (HLA-DRB1*01:01:01G) model.

	Dependent Variable: Outcome
Age	−0.06 *** (0.02)
SexMale	−1.71 *** (2.07)
HLA-DRB1*01:01:01G-carriage	1.1 ** (0.45)
Age:SexMale	−0.04 (0.03)
Constant	3.7 *** (1.16)
Observation	183
Log Likelihood	−101.49
Akaike Inf. Crit	212.98

Note: ** *p* < 0.05; *** *p* < 0.01.

**Table 14:** Coefficients for ”Best” model by AIC for locus DQB1.

	Dependent Variable: Outcome
Age	−0.08 *** (0.02)
SexMale	−1.1 *** (0.39)
HLA-DQB1*04:01:01G-carriage	−16.68 (882.74)
HLA-DQB1*05:01:01G-carriage	0.99 ** (0.41)
HLA-DQB1*05:03:01G-carriage	1.08 (0.66)
Constant	5.14 *** (1.05)
Observation	183
Log Likelihood	−99.25
Akaike Inf. Crit	210.5

Note: ** *p* < 0.05; *** *p* < 0.01.

**Table 15:** Coefficients for one-allele (HLA-DQB1*05:01:01G) model.

	Dependent Variable: Outcome
Age	−0.06 *** (0.02)
SexMale	1.69 (2.02)
HLA-DQB1*05:01:01G-carriage	0.94 ** (0.40)
Age:SexMale	−0.04 (0.03)
Constant	3.68 *** (1.16)
Observation	183
Log Likelihood	−101.77
Akaike Inf. Crit	213.53

Note: ** *p* < 0.05; *** *p* < 0.01.

**Table 16:** Coefficients of «best» model by AIC with HLA allele, which were statistically significant at locus models.

	Dependent Variable: Outcome
Age	−0.06 *** (0.02)
SexMale	1.96 ** (2.08)
HLA-C*08:02:01G-carriage	1.91 ** (0.85)
HLA-DRB1*01:01:01G-carriage	1.1 ** (0.47)
Age:SexMale	−0.05 (0.03)
Constant	3.82 *** (1.18)
Observation	183
Log Likelihood	−98.76
Akaike Inf. Crit	209.53

Note: ** *p* < 0.05; *** *p* < 0.01.

**Table 17:** List of the groups subjected to the analysis.

Group Name	Designation	Number
Control group (participants of the bone marrow donor registry)	1	475
Recovered patients with severe symptoms	2	76
Recovered patients with severe symptoms (under 65 y.o.)	2A	30
Recovered patients with severe symptoms (above 65 y.o.)	2B	46
Deceased patients	3	157
Deceased patients (under 65 y.o.)	3A	74
Deceased patients (above 65 y.o.)	3B	33

**Table 18:** Demographic data in Group 1 patients with COVID-19.

Group 1 (Control Group)	Aged 65 y.o. or Below
The number of patients	total 475
Age, median, Q25–Q75	21 (19–25)
Sex	
Female	338
Male	137

**Table 19:** Demographic data in Group 2 patients with COVID-19.

Group 2 (Recovered)	Aged 65 y.o. or Below	Aged Above 65 y.o.
The number of patients	46	30
Age, median (Q25–Q75)	56 (47–59)	72 (69–78)
Sex		
Female	24	25
Male	22	5

**Table 20:** Demographic data in Group 3 patients with COVID-19.

Group 3 (Deceased)	Aged 65 y.o. or Below	Aged Above 65 y.o.
The number of patients	33	74
Age, median (Q25–Q75)	58 (54–62)	81 (75–86)
Sex		
Female	15	45
Male	18	29

**Table 21:** The example of using two methods for collecting patient’s data to produce a contingency matrix of an A gene.

Selection Principle	The Example of the Data	Freedom Degrees
The joint analysis of an allele pair (V1).	‘24:02_68:24’, ‘25:01_68:02’, ‘03:01_30:04’, ‘24:02_32:01’, ‘01:01_02:01’, ‘01:01_25:01’, ‘01:02_29:02’, ‘02:01_30:01’, ‘33:01_68:01’, ‘25:01_33:01’, ‘03:02_68:01’, ‘01:01_29:02’, …. ‘02:05_11:01’, ‘01:01_68:01’, ‘23:01_23:01’, ‘03:01_29:02’, ‘02:01_24:02’, ‘03:01_33:03’, ‘24:02_29:02’, ‘02:01_68:01’, ‘33:01_68:02’, ‘03:01_23:01’, ‘24:02_24:02’, ‘25:01_32:01’, ‘11:01_30:01’, ‘01:01_03:01’, ‘02:01_30:04’, ‘23:01_30:01’	337
Two alleles separately analyzed based on the number of carriers (V2)	‘26:01’, ‘30:02’, ‘69:01’, ‘25:01’, ‘30:01’, ‘31:01’, ‘68:02’, ‘02:17’, ‘01:01’, ‘23:01’, ‘33:01’, ‘02:05’, ‘01:03’, ‘02:01’, ‘33:03’, ‘24:03’, ‘29:02’, ‘32:01’, ‘30:04’, ‘03:02’, ‘29:01’, ‘02:07’, ‘11:01’, ‘66:01’, ‘68:01’, ‘68:24’, ‘03:01’, ‘02:06’, ‘24:02’, ‘01:02’	46

**Table 22:** Comparison of the obtained alleles in the context of COVID-19 based on the pertinent literature.

Paper	Comment (Population)	A*	B*	C*	DRB1*	DQB1*	DPB1*
**Our results**	Comparison of deceased and recovered patients with population data (Russian)		14:02	08:02	01:01 01:02	05:03 05:01	
	Haplotypes	B*14:02:01G~C*08:02:01G A*02:01:01G~B*27:02:01G~C*02:02:02G DRB1*01:01:01G~DQB1*05:01:01G ~DPB1*04:02:01G DRB1*07:01:01G~DQB1*02:01:01G ~DPB1*17:01:01G					
SARS HLA associations							
**[33]**	The association between HLA genes (Low typing resolution) and SARS (Chinese)	26			04, 16, 09		
**[34]**	Analysis of SARS severity in patients and high risk health care workers groups (Taiwanese)		46:01 54:01 13:01				
**[35]**	The association between SARS and HLA alleles (Chinese)		07:03		03:01	06:01	
COVID-19 HLA associations							
**[7]**	Patients with severe symptoms compared with mild ones (Chinese)	11:01	51:01 13:02	14:02	14:04 01:01 12:01		03:01
**[15]**	Vulnerable/persistent to COVID-19 in silico (805 distinct populations)	02:02 25:01	46:01 15:03	01:02 12:03			
**[16]**	Binding affinity of the HLA proteins to the SARS-CoV-2 proteome (10 groups including different populations)	02:01 24:03 68:02		14:02 14:03	01:02 07:01		
**[26]**	HLA allele frequencies in 82 humans with COVID-19 (Chinese)		15:27	07:29			
**[24]**	Correlation between HLA haplotypes and COVID-19 (Italian)	01:01 02:01	18:01 08:01	07:01 07:01	03:01 11:04		
**[18]**	The influence of HLA genotype on COVID-19 severity (North European)				04:01		
**[17]**	The in silico correlation between COVID-19 fatality and HLA (Mexican)				01:01		
**[27]**	Regression analysis of mortality across 74 countries (Japan, China, Russia etc.)			05			
**[21]**	The in silico association between HLA and COVID-19 mortality in 19 countries (Thailand, England, USA, Japan etc.)	02:01 11:01 24:02					
**[36]**	The association susceptibility and severity of COVID-19 review	25:01 02:02	15:27 46:01 15:03	01:02 07:29 12:03			
**[23]**	The in silico retrospective prediction of HLA for COVID-19 prognosis (American)	11:01		04:01			

## Data Availability

The data described in this article are openly available in the Supplementary material.

## Supplementary Materials

The following supporting information can be downloaded at: https://www.mdpi.com/article/10.3390/ijms24043068/s1, Table S1: HLA-genotype for all samples; Table S2a: Estimated frequency for six HLA loci; Table S2b: Estimated frequency for five HLA loci; Table S2c: Estimated frequency for I class HLA loci; Table S2d: Estimated frequency for II class HLA loci; Table S2e: Estimated frequency for HLA-B and HLA-C loci; Table S3: Group combinations for Chi-square test; Table S4: Significant alleles (Exact Fisher’s test).

## References

[1] Meyerowitz-Katz G., Merone L. (2020). A Systematic Review and Meta-Analysis of Published Research Data on COVID-19 Infection Fatality Rates. Int. J. Infect. Dis..

[2] (2023). World Health Organization COVID-19 Weekly Epidemiological Update. World Heal. Organ..

[3] Mao R., Qiu Y., He J.S., Tan J.Y., Li X.H., Liang J., Shen J., Zhu L.R., Chen Y., Iacucci M. (2020). Manifestations and Prognosis of Gastrointestinal and Liver Involvement in Patients with COVID-19: A Systematic Review and Meta-Analysis. Lancet Gastroenterol. Hepatol..

[4] Spinato G., Fabbris C., Polesel J., Cazzador D., Borsetto D., Hopkins C., Boscolo-Rizzo P. (2020). Alterations in Smell or Taste in Mildly Symptomatic Outpatients With SARS-CoV-2 Infection. JAMA.

[5] Vetter P., Vu D.L., L’Huillier A.G., Schibler M., Kaiser L., Jacquerioz F. (2020). Clinical Features of COVID-19. BMJ.

[6] Zhou F., Yu T., Du R., Fan G., Liu Y., Liu Z., Xiang J., Wang Y., Song B., Gu X. (2020). Clinical Course and Risk Factors for Mortality of Adult Inpatients with COVID-19 in Wuhan, China: A Retrospective Cohort Study. Lancet.

[7] Wang F., Huang S., Gao R., Zhou Y., Lai C., Li Z., Xian W., Qian X., Li Z., Huang Y. (2020). Initial Whole-Genome Sequencing and Analysis of the Host Genetic Contribution to COVID-19 Severity and Susceptibility. Cell Discov..

[8] (2020). The Severe Covid-19 GWAS Group Genomewide Association Study of Severe Covid-19 with Respiratory Failure. N. Engl. J. Med..

[9] Cascella M., Rajnik M., Aleem A., Dulebohn S.C., Napoli R.D., Sivoravong J.C., Burkhardt C. (2022). Features, Evaluation, and Treatment of Coronavirus Disease.

[10] Dutta M., Dutta P., Medhi S., Borkakoty B., Biswas D. (2018). Polymorphism of HLA Class I and Class II Alleles in Influenza A(H1N1)Pdm09 Virus Infected Population of Assam, Northeast India. J. Med. Virol..

[11] Stephens H.A.F. (2010). HLA and Other Gene Associations with Dengue Disease Severity. Curr. Top. Microbiol. Immunol..

[12] Teixeira S.L.M., De Sá N.B.R., Campos D.P., Coelho A.B., Guimarães M.L., Leite T.C.N.F., Veloso V.G., Morgado M.G. (2014). Association of the HLA-B*52 Allele with Non-Progression to AIDS in Brazilian HIV-1-Infected Individuals. Genes Immun..

[13] Ding S.J., Zhang Y., Zhang X.M., Jiang X.L., Pang B., Song Y.H., Wang J.X., Pei Y.W., Zhu C.F., Wang X.J. (2016). Correlation Between HLA-A, B and DRB1 Alleles and Severe Fever with Thrombocytopenia Syndrome. PLoS Negl. Trop. Dis..

[14] Goverdhan S.V., Howell M.W., Mullins R.F., Osmond C., Hodgkins P.R., Self J., Avery K., Lotery A.J. (2005). Association of HLA Class I and Class II Polymorphisms with Age-Related Macular Degeneration. Investig. Ophthalmol. Vis. Sci..

[15] Nguyen A., David J.K., Maden S.K., Wood M.A., Weeder B.R., Nellore A., Thompson R.F. (2020). Human Leukocyte Antigen Susceptibility Map for Severe Acute Respiratory Syndrome Coronavirus 2. J. Virol..

[16] Barquera R., Collen E., Di D., Buhler S., Teixeira J., Llamas B., Nunes J.M., Sanchez-Mazas A. (2020). Binding Affinities of 438 HLA Proteins to Complete Proteomes of Seven Pandemic Viruses and Distributions of Strongest and Weakest HLA Peptide Binders in Populations Worldwide. Hla.

[17] Romero-López J.P., Carnalla-Cortés M., Pacheco-Olvera D.L., Ocampo-Godínez J.M., Oliva-Ramírez J., Moreno-Manjón J., Bernal-Alferes B., López-Olmedo N., García-Latorre E., Domínguez-López M.L. (2021). A Bioinformatic Prediction of Antigen Presentation from SARS-CoV-2 Spike Protein Revealed a Theoretical Correlation of HLA-DRB1*01 with COVID-19 Fatality in Mexican Population: An Ecological Approach. J. Med. Virol..

[18] Langton D.J., Bourke S.C., Lie B.A., Reiff G., Natu S., Darlay R., Burn J., Echevarria C. (2021). The Influence of HLA Genotype on the Severity of COVID-19 Infection. Hla.

[19] Shkurnikov M., Nersisyan S., Jankevic T., Galatenko A., Gordeev I., Vechorko V., Tonevitsky A. (2021). Association of HLA Class I Genotypes With Severity of Coronavirus Disease-19. Front. Immunol..

[20] Sanchez-Mazas A. (2020). HLA Studies in the Context of Coronavirus Outbreaks. Swiss Med. Wkly..

[21] Tomita Y., Ikeda T., Sato R., Sakagami T. (2020). Association between HLA Gene Polymorphisms and Mortality of COVID-19: An in Silico Analysis. Immun. Inflamm. Dis..

[22] Iturrieta-Zuazo I., Rita C.G., García-Soidán A., de Malet Pintos-Fonseca A., Alonso-Alarcón N., Pariente-Rodríguez R., Tejeda-Velarde A., Serrano-Villar S., Castañer-Alabau J.L., Nieto-Gañán I. (2020). Possible Role of HLA Class-I Genotype in SARS-CoV-2 Infection and Progression: A Pilot Study in a Cohort of Covid-19 Spanish Patients. Clin. Immunol..

[23] Warren R.L., Birol I. (2021). HLA alleles measured from COVID-19 patient transcriptomes reveal associations with disease prognosis in a New York cohort. PeerJ.

[24] Pisanti S., Deelen J., Gallina A.M., Caputo M., Citro M., Abate M., Sacchi N., Vecchione C., Martinelli R. (2020). Correlation of the Two Most Frequent HLA Haplotypes in the Italian Population to the Differential Regional Incidence of Covid-19. J. Transl. Med..

[25] Warren R.L., Birol I. (2021). HLA Predictions from the Bronchoalveolar Lavage Fluid and Blood Samples of Eight COVID-19 Patients at the Pandemic Onset. Bioinformatics.

[26] Wang W., Zhang W., Zhang J., He J., Zhu F. (2020). Distribution of HLA Allele Frequencies in 82 Chinese Individuals with Coronavirus Disease-2019 (COVID-19). Hla.

[27] Sakuraba A., Haider H., Sato T. (2020). Population Difference in Allele Frequency of HLA-C*05 and Its Correlation with Covid-19 Mortality. Viruses.

[28] Beerli P. (2005). Pairwise Distance Methods. Comput. Evol. Biol..

[29] Holsinger K.E., Weir B.S. (2009). Genetics in Geographically Structured Populations: Defining, Estimating and Interpreting FST. Nat. Rev. Genet..

[30] Robinson J., Barker D.J., Georgiou X., Cooper M.A., Flicek P., Marsh S.G.E. (2020). IPD-IMGT/HLA Database. Nucleic Acids Res..

[31] Excoffier L., Lischer H.E.L. (2010). Arlequin Suite Ver 3.5: A New Series of Programs to Perform Population Genetics Analyses under Linux and Windows. Mol. Ecol. Resour..

[32] Aickin M., Gensler H. (1996). Adjusting for Multiple Testing When Reporting Research Results: The Bonferroni vs. Holm Methods. Am. J. Public Health.

[33] Liu D., Qiu Y., Zha Y., Li W., Li D., Wu T. (2018). Association of HLA Class Ⅰ and Class Ⅱ Genes with Severe Acute Respiratory Syndrome in the Northern Chinese Population. Blood Genom..

[34] Lin M., Tseng H.K., Trejaut J.A., Lee H.L., Loo J.H., Chu C.C., Chen P.J., Su Y.W., Lim K.H., Tsai Z.U. (2003). Association of HLA Class I with Severe Acute Respiratory Syndrome Coronavirus Infection. BMC Med. Genet..

[35] Ng M.H.L., Lau K., Li L., Cheng S., Chan W.Y., Hui P.K., Zee B., Leung C., Sung J.J.Y. (2004). Association of Human-Leukocyte-Antigen Class I (B*0703) and Class II (DRB1*0301) Genotypes with Susceptibility and Resistance to the Development of Severe Acute Respiratory Syndrome. J. Infect. Dis..

[36] Fricke-Galindo I., Falfán-Valencia R. (2021). Genetics Insight for COVID-19 Susceptibility and Severity: A Review. Front. Immunol..

