# Supplementary figures and images for: The Search of Association of HLA Class I and Class II Alleles with COVID-19 Mortality in the Russian Cohort
Source: Int J Mol Sci. 2023 Feb 4;24(4):3068. doi: 10.3390/ijms24043068 (PMC9960097; doi:10.3390/ijms24043068)

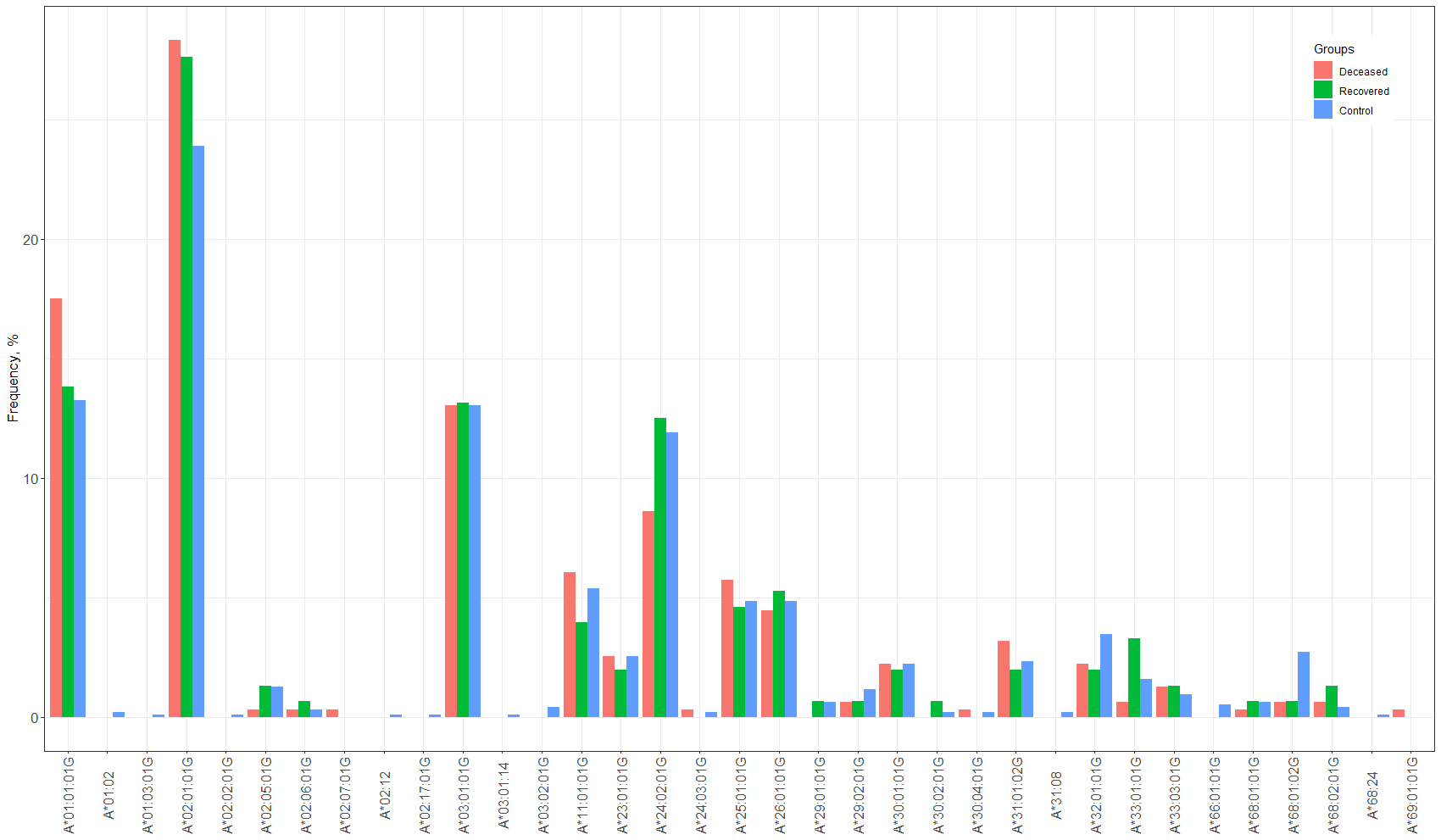

Supplement: Supplementary file 1 [file ijms-24-03068-s001.zip › Figure S1a. HLA-A_allele_freq.png]

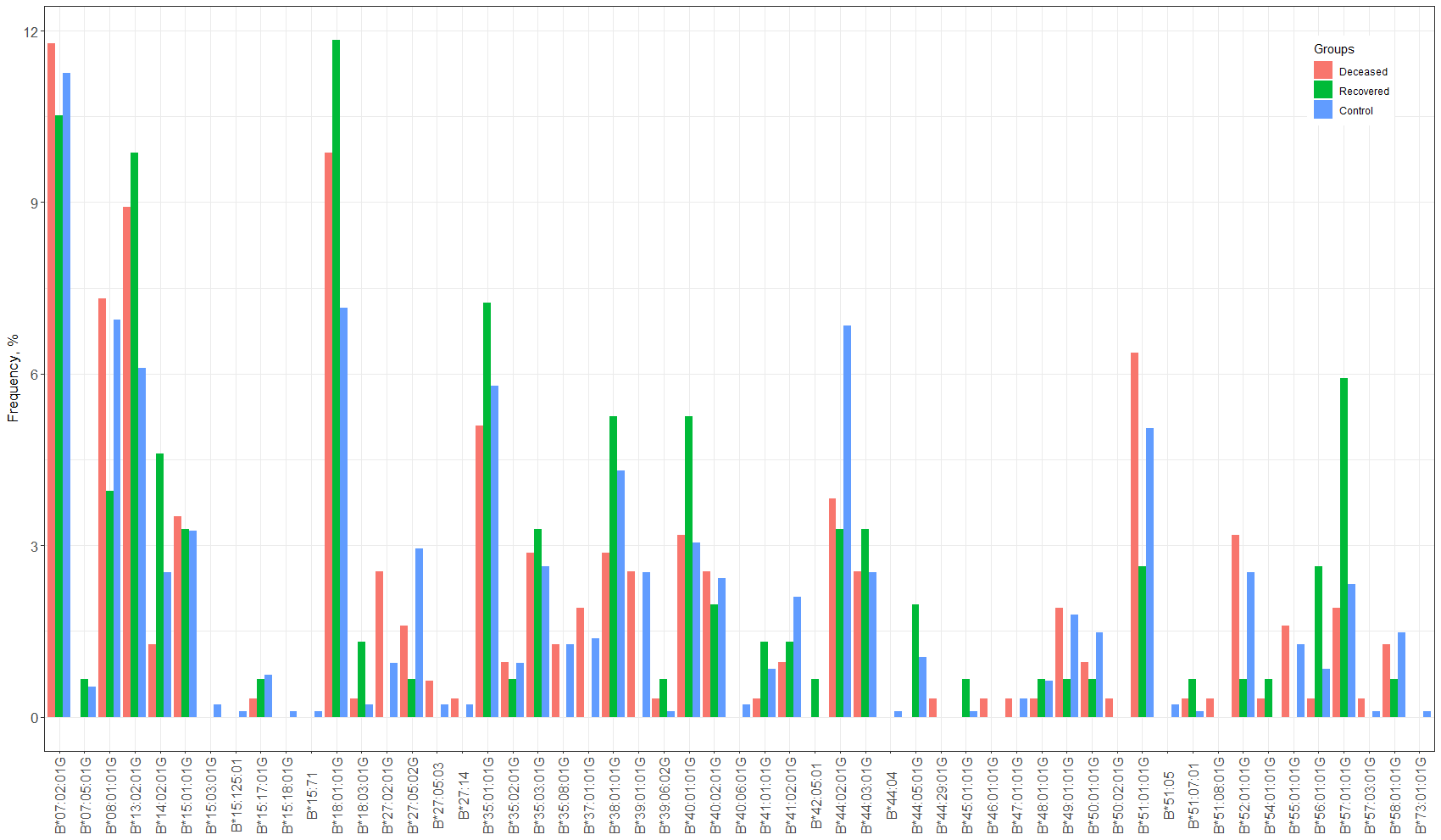

Supplement: Supplementary file 1 [file ijms-24-03068-s001.zip › Figure S1b. HLA-B_allele_freq.png]

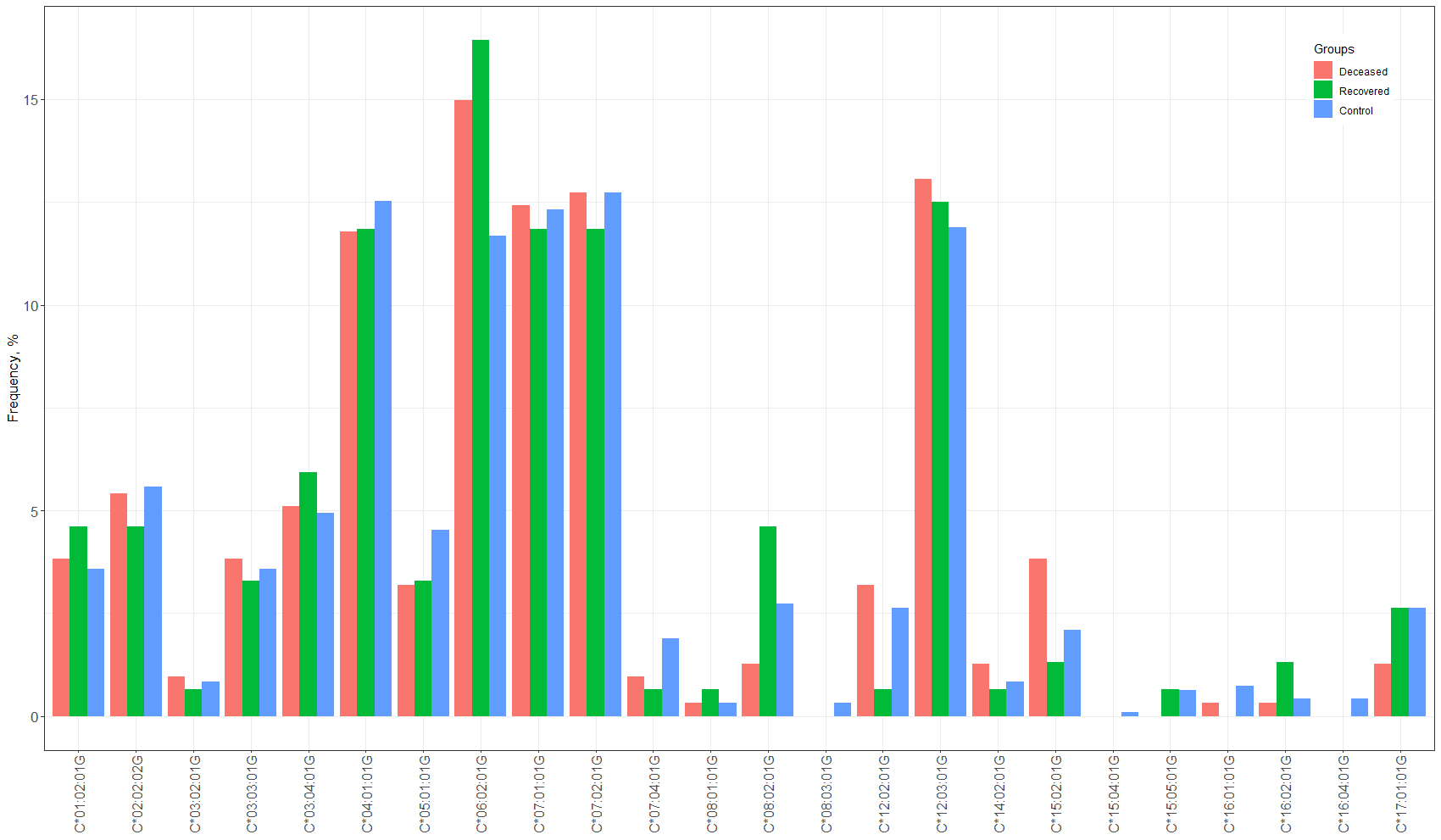

Supplement: Supplementary file 1 [file ijms-24-03068-s001.zip › Figure S1c. HLA-C_allele_freq.png]

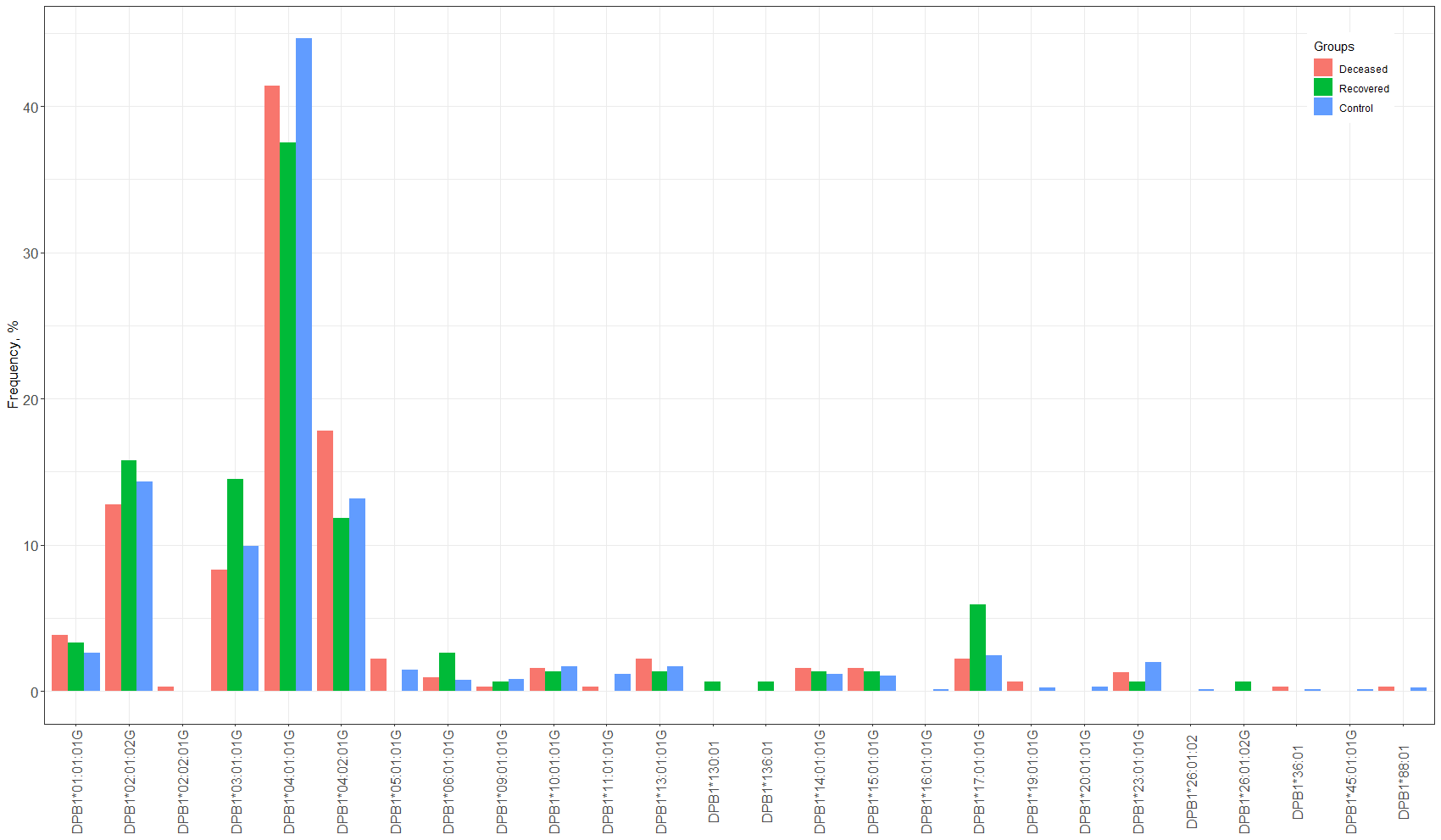

Supplement: Supplementary file 1 [file ijms-24-03068-s001.zip › Figure S1d. HLA-DPB1_allele_freq.png]

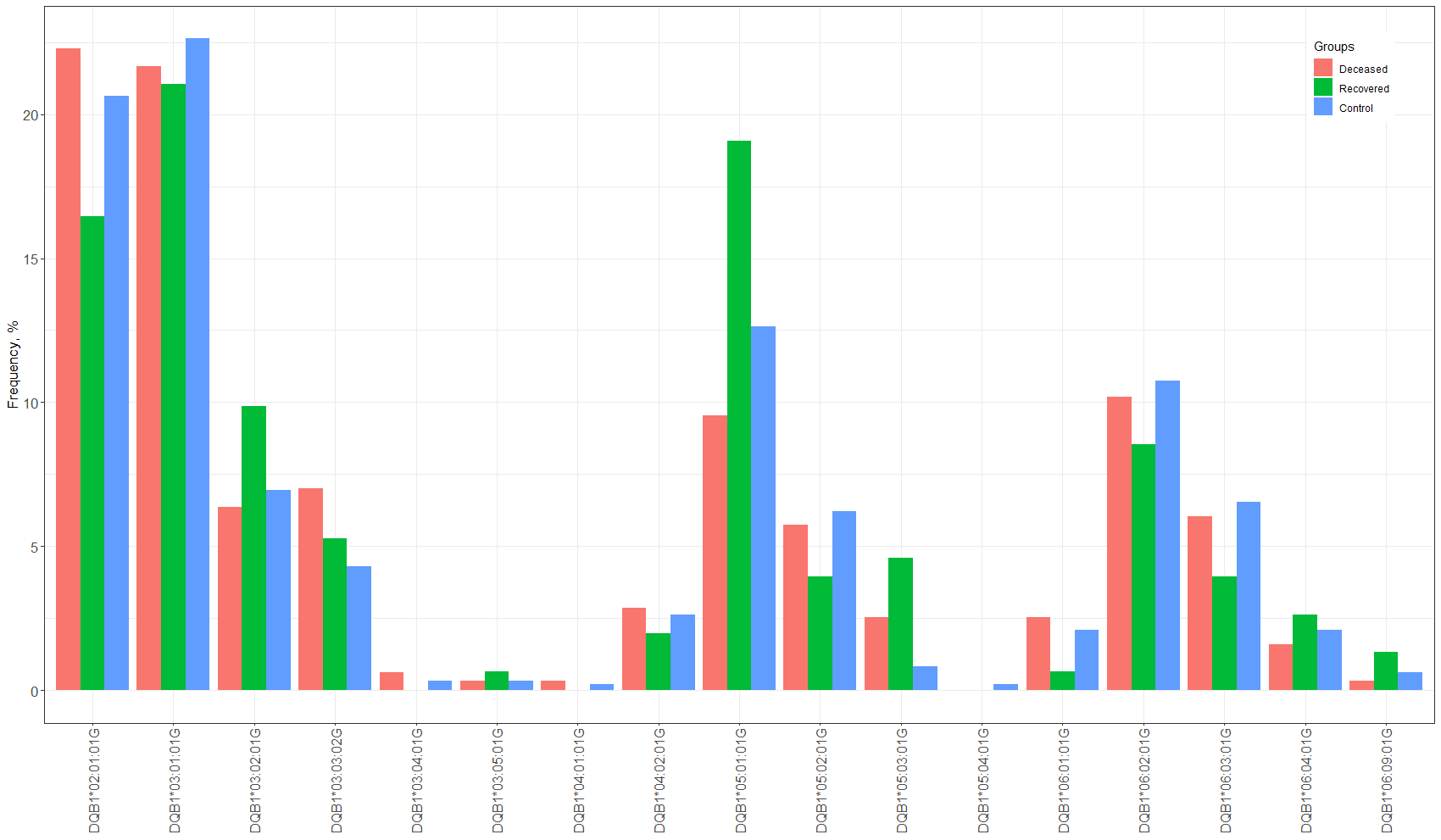

Supplement: Supplementary file 1 [file ijms-24-03068-s001.zip › Figure S1e. HLA-DQB1_allele_freq.png]

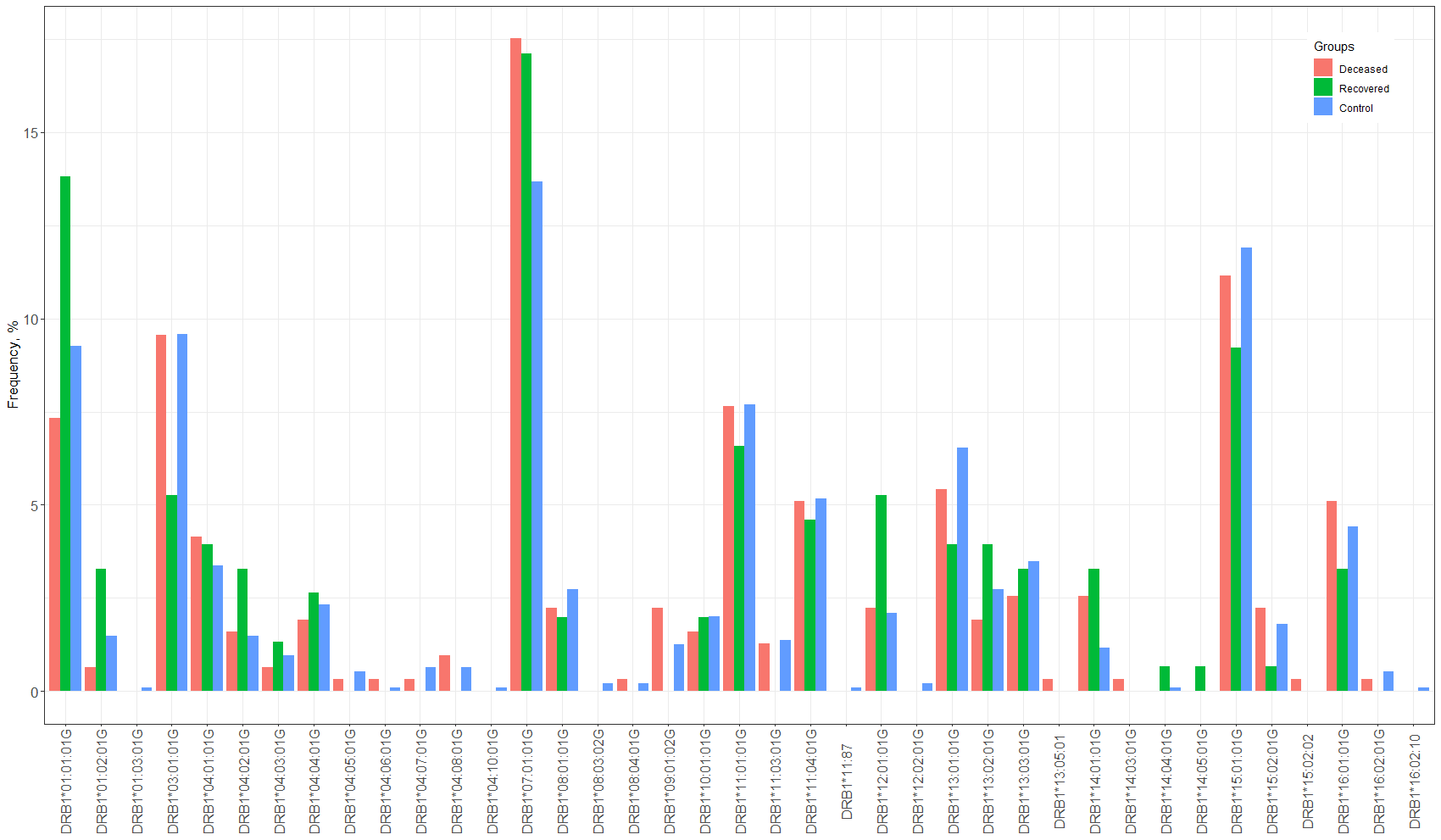

Supplement: Supplementary file 1 [file ijms-24-03068-s001.zip › Figure S1f. HLA-DRB1_allele_freq.png]
